# Identification and Functional Analysis of PANoptosis‐Associated Genes in the Progression From Sepsis to ARDS

**DOI:** 10.1002/iid3.70136

**Published:** 2025-01-24

**Authors:** Zhong‐Hua Lu, Yan Tang, Hu Chen, Feng Liu, Mei Liu, Lu Fu, Xian‐Kai Wang, Ming‐Juan Li, Wei‐Li Yu, Yun Sun

**Affiliations:** ^1^ The First Department of Critical Care Medicine The Second Affiliated Hospital of Anhui Medical University Hefei Anhui China; ^2^ Department of Rehabilitation Medicine The Second Affiliated Hospital of Anhui Medical University Hefei Anhui China; ^3^ Department of Critical Care Medicine The First Affiliated Hospital of Chongqing Medical University Chongqing City China

**Keywords:** ARDS, NDRG1, PANoptosis, sepsis

## Abstract

**Background:**

Sepsis and acute respiratory distress syndrome (ARDS) are common inflammatory conditions in intensive care, with ARDS significantly increasing mortality in septic patients. PANoptosis, a newly discovered form of programmed cell death involving multiple cell death pathways, plays a critical role in inflammatory diseases. This study aims to elucidate the PANoptosis‐related genes (PRGs) and their involvement in the progression of sepsis to ARDS.

**Methods:**

This study analyzed differentially expressed genes (DEGs) associated with PRGs to explore their role in the progression of immune disorders from sepsis to septic ARDS. A diagnostic prediction model was constructed based on key PRGs identified through bioinformatics analysis. Functional enrichment analyses were conducted to determine pathway involvement, and correlations with immune cells were assessed. Mendelian randomization analysis was applied to investigate potential causal links between specific PRGs and ARDS. Immunohistochemical analysis was used to evaluate PRG expression in lung tissue.

**Results:**

The prediction model effectively distinguished septic ARDS patients from those with sepsis. NDRG1 expression was elevated in ARDS, while DDX3X, PTPRC, and TNFSF8 were downregulated. NDRG1 showed a positive correlation with activated dendritic cells, whereas DDX3X, PTPRC, and TNFSF8 were positively associated with neutrophils and negatively correlated with CD56bright NK cells. Functional enrichment analysis highlighted spliceosome function, MAPK signaling, endocytosis, and antigen processing pathways as significantly associated with these PRGs. Mendelian randomization suggested a causal link between NDRG1 and ARDS, and immunohistochemical analysis revealed its predominant expression near vascular walls. In a mouse model of sepsis, suppression of NDRG1 alleviated lung injury.

**Conclusion:**

PANoptosis may contribute to immune dysregulation in sepsis‐associated ARDS. NDRG1 is identified as a potential therapeutic target, offering new avenues for mitigating ARDS progression and improving patient outcomes.

## Graphical abstract

This study identifies four key PANoptosis‐related genes (NDRG1, DDX3X, PTPRC, and TNFSF8) that are differentially expressed in sepsis and septic ARDS. The prediction model incorporating these genes shows high diagnostic accuracy, with NDRG1 emerging as a potential therapeutic target due to its significant expression and correlation with immune cells and pathways involved in ARDS progression.

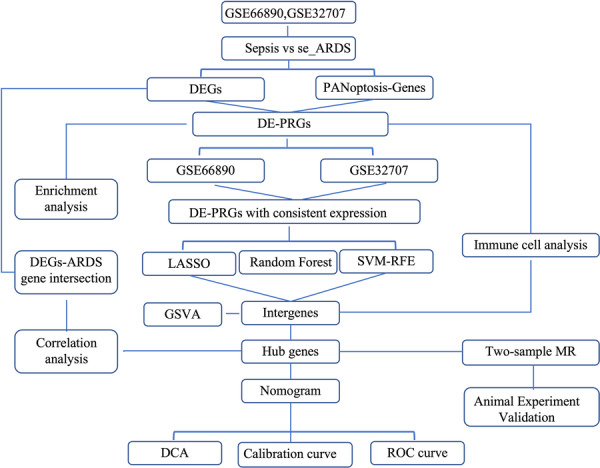

## 1 Introduction

Sepsis significantly contributes to negative outcomes in ICU patients, with sepsis‐related deaths comprising 19.7% of global fatalities [1]. The onset of acute respiratory distress syndrome (ARDS) in these patients increases the risk of death during ICU and hospital stays [2]. Despite extensive research over the years, a definitive treatment for ARDS has yet to be developed. Existing interventions, including protective lung ventilation, prone ventilation, and negative fluid balance, mainly offer supportive care [3]. Diagnosis relies on functional methods like SpO_2_/FiO_2_, the oxygenation index, or physician judgment due to resource limitations, as per the 2023 global definition [4, 5].

The absence of precise early diagnostic markers based on pathogenesis highlights the urgent need for innovative interventions and improved management strategies to enhance patient outcomes.

Addressing septic ARDS challenges may benefit from effective biomarkers, targets, and innovative treatments, particularly by targeting cell death pathways. Programmed cell death (PCD), including pyroptosis, necroptosis, and apoptosis, is crucial for maintaining organismal balance [6, 7]. PANoptosis, which integrates these pathways, represents a key genetic and molecular feature. Although limited evidence exists on PANoptosis role in ARDS, these PCD pathways are associated with acute lung injury [8, 9, 10, 11, 12]. For example, CD95/CD95 ligand upregulation triggers epithelial cell apoptosis, while LPS induces NET formation, leading to alveolar macrophage pyroptosis and inflammation in ARDS [12, 13, 14]. Additionally, Wang et al. showed that RIP3‐mediated necroptosis plays a role in LPS‐induced inflammation and acute lung injury in mice [15]. PANoptosis is linked to ARDS development and the immune cell‐driven inflammatory response [16, 17]. Although the roles of pyroptosis, apoptosis, and necrosis in ARDS progression through different immunomodulatory pathways have been studied separately, research on PANoptosis's impact and its relationship with ARDS is still limited.

Our study utilized bioinformatics analysis to assess the PANoptosis signature and its correlation with the onset of septic ARDS, as well as to explore its diagnostic potential. Concentrating on the progression from sepsis to septic ARDS in critically ill patients, our objective was to identify specific genes implicated in this transition. Recognizing that sepsis‐induced ARDS triggers unique inflammatory pathways, comparisons with healthy controls were deemed inappropriate for elucidating the distinct mechanisms involved. Therefore, consistent with other research conducted in critical care environments, our analysis focused on comparisons within diseased cohorts to facilitate the identification of relevant factors.

## 2 Materials and Methods

### 2.1 Data Collection and Download

Figure S1 illustrates the bioinformatics analysis strategy. ARDS gene expression data sets GSE66890 and GSE32707 were obtained from the GEO database. The GSE66890 data set comprises 28 sepsis and 29 sepsis‐related ARDS samples, collected using the HuGene‐1_0‐st Affymetrix Human Gene 1.0 ST Array platform (GPL6244). The GSE32707 data set includes 28 sepsis samples and 13 sepsis‐related ARDS samples, derived from the Illumina HumanHT‐12 V4.0 expression beadchip platform (GPL10558). To examine differential gene expression related to ARDS progression from a baseline of sepsis, we selected data sets focused on critically ill sepsis and ARDS patients. Given this focus, healthy controls were not included, as the study aimed to capture sepsis‐specific progression dynamics. This approach aligns with prior research emphasizing disease progression analysis within critically ill cohorts [18].

### 2.2 Identification of Differentially Expressed PANoptosis‐Related Genes (DE‐PRGs)

We used the “limma” package in R (v4.2.2) to analyze differential gene expression between septic ARDS and sepsis patients in the GSE66890 and GSE32707 cohorts, with criteria of |logFC| ≠ 0 and adj.P.Val < 0.05 using the false discovery rate (FDR) method for multiple testing correction. Eighteen PRGs with a relevance score above 3 were obtained from the GeneCards database. Genes that overlapped between differentially expressed genes (DEGs) and PRGs, referred to as DE‐PRGs, were selected for further analysis.

### 2.3 Analyses of Functional Enrichment

Functional enrichment and clustering of DE‐PRGs were performed using the “clusterProfiler” R package with GO and KEGG analyses, considering *p* and *q* values < 0.05 for significance. The Metascape database was also used to explore related functions, with significance marked by at least three overlaps and a *p* < 0.01 (https://metascape.org/). Gene sets from the molecular signature database were analyzed for key biological functions using the gene set variation analysis (GSVA) algorithm.

### 2.4 Model Construction and Identification of Key Genes

The study used three machine learning algorithms—LASSO, SVM‐RFE, and RF—to identify key diagnostic or prognostic variables. LASSO, via the “glmnet” R package, employs regularization for variable selection [19]. SVM‐RFE, using the “e1071” package, is a popular method for classification and regression, applied here to pinpoint genes with significant discriminant abilities [20]. The RF algorithm, utilizing ensemble learning to enhance accuracy, applied the “randomForest” package to refine the selection of candidate biomarkers [21]. Key genes for developing diagnostic models for sepsis‐related ARDS were identified as those common to all three machine learning algorithms. The model's predictive performance was assessed with receiver operating characteristic (ROC) curve analysis.

### 2.5 Immune Infiltration Analysis

We used the “GSVA” R package to analyze normalized septic ARDS gene expression data against a 782‐gene set designed to predict the abundance of 28 tumor‐infiltrating immune cells (TIICs) based on shared biological functions, chromosomal locations, and physiological regulation [22]. The septic ARDS GEP data were normalized and assessed for TIICs enrichment in the blood of septic ARDS patients.

### 2.6 Unraveling the Genetic Influence on ARDS Susceptibility Using Expression Quantitative Trait Loci (eQTL)‐Based Mendelian Randomization

eQTLs are genetic loci influencing gene expression. Using eQTLs as instrumental variables allows for the investigation of phenotype changes due to gene expression variations. This study employed eQTL data for key genes from the MRC Integrative Epidemiology Unit's IEU Open GWAS database and ARDS GWAS data from the R10 release of the FinnGen consortium. A 2‐sample Mendelian randomization approach was applied using R software to explore the causal relationship between key upregulated genes and ARDS onset. The inverse variance weighted (IVW) method, weighted median (WM), and simple median (SM) confirmed the relationship, with Cochran's *Q* test evaluating heterogeneity. An MR‐Egger intercept test assessed potential pleiotropy by examining the regression intercept's *p*‐value for horizontal pleiotropic effects. A leave‐one‐out (LOO) sensitivity analysis evaluated each SNP's impact on the overall IVW estimate. The results are presented as odds ratios (OR) and accompanied by 95% confidence intervals (CI), with statistical significance determined by a *p*‐value less than 0.05.

### 2.7 The Construction of Septic ARDS Models

To create a CLP‐induced sepsis ARDS model in mice, anesthesia was administered via intraperitoneal injection of pentobarbital. The procedure involved shaving, disinfecting, making an incision, exposing the cecum, ligating, and puncturing it with a 22‐gauge needle. Fecal matter was removed from the puncture site before closing the incision. ARDS‐Sham mice underwent the same steps, excluding cecal ligation. In the EMD638683 (NDRG1 inhibitor, MedChemExpress, Cat# HY‐15193) treatment group, septic mice were administered EMD638683 intraperitoneally at a dosage of 10 mg/kg, given 12 h before inducing the sepsis model. For the EMD638683 control group, mice received an equivalent volume of DMSO at the corresponding time. All mice were euthanized 24 h after the procedure. The study received approval from the Anhui Medical University Experimental Animal Ethics Committee (LLSC20240738) and adhered to their guidelines.

### 2.8 Lung Histopathology and Immunohistochemical Staining

The right superior lobe was embedded in paraffin, sectioned into 5 μm slices, and subjected to hematoxylin and eosin staining. The presence of edema, inflammation, hemorrhage, atelectasis, necrosis, and hyaline membrane formation was evaluated and scored on a scale from 0 to 4 to determine the total lung injury score [23]. The tissue sections were deparaffinized, rehydrated, underwent antigen retrieval, and were subsequently blocked with bovine serum albumin (BSA) for 1 h prior to an overnight incubation at 4°C with primary antibodies (NDRG1, CST, RatmAb #5196S). The sections were incubated overnight at 4°C with NDRG1 primary antibodies (1:2000 dilution), washed three times with PBS, and then treated with a 1:200 dilution of biotinylated secondary antibody (HRP‐conjugated goat anti‐rabbit, Scrviccbio, RatmAb #GB23303). Visualization was done using diaminobenzidine (DAB, China) and hematoxylin, and images were analyzed at ×200 magnification with a light microscope.

### 2.9 Statistical Analysis

Bioinformatics analyses were performed using R software (version 4.2.2). For animal experiments, data were analyzed in GraphPad Prism (version 9.5.1) and presented as mean ± standard deviation (SD). Comparisons between two groups were conducted using an unpaired two‐tailed Student's *t*‐test for normally distributed data or the Mann−Whitney *U* test for non‐normally distributed data, with statistical significance defined as *p* < 0.05.

## 3 Results

### 3.1 Screening of DE‐PRGs in Patients With Sepsis and Septic ARDS

**Figure 1:**
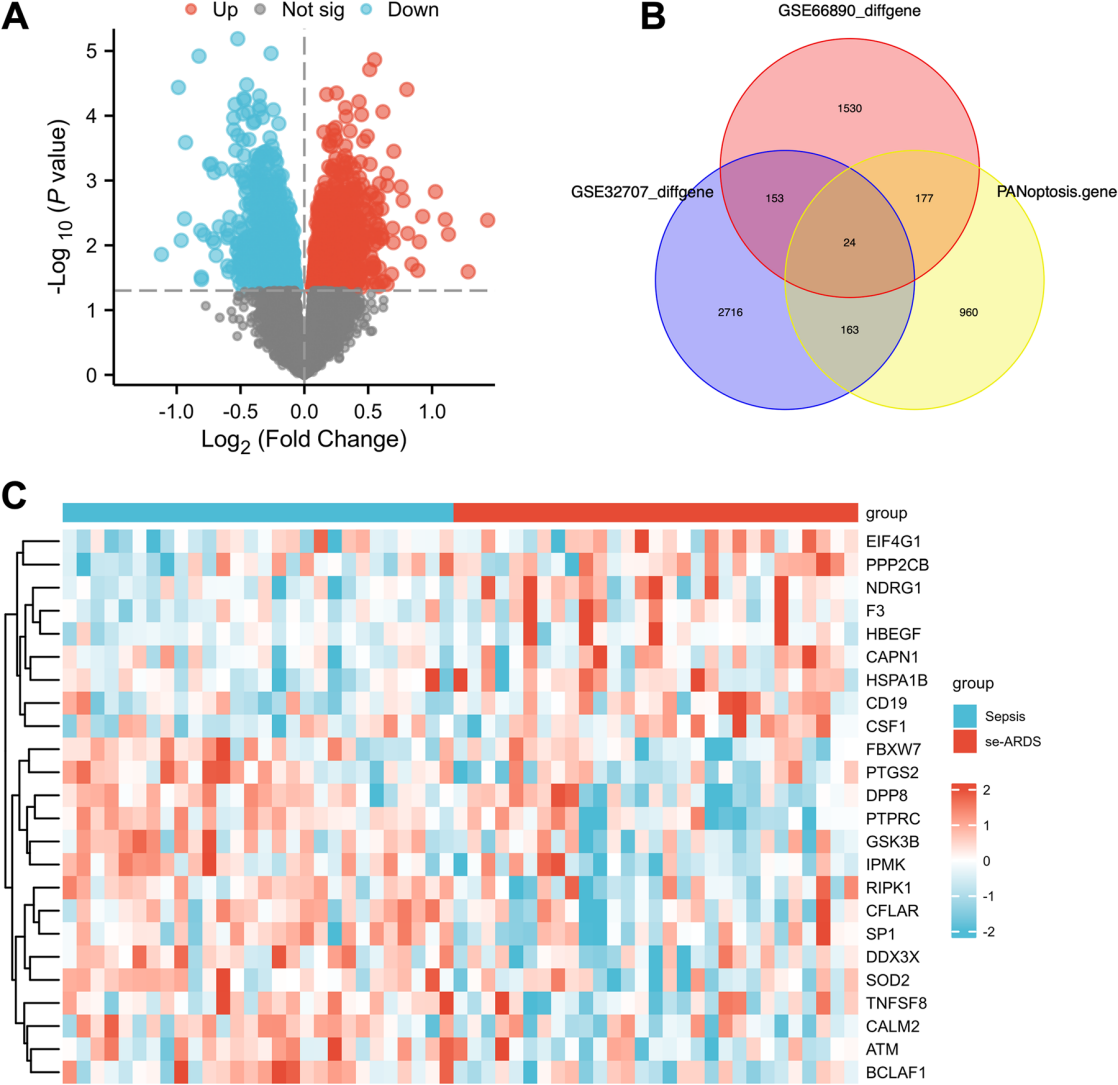
Identification of differentially expressed PANoptosis‐related genes (DE‐PRGs). (A) Volcano plot displaying the DEGs, with fold change and *p*‐value as axes. Upregulated genes are marked with red dots, downregulated genes with blue dots, and nonsignificant genes with gray dots. (B) Venn diagram illustrating the overlap of DEGs identified in data sets GSE66890 and GSE32707 with known PANoptosis‐related genes (PRGs). (C) Heatmap representing the expression patterns of 24 identified DE‐PRGs.

To investigate the role of PANoptosis in the progression from sepsis to septic ARDS, we began by identifying DEGs related to PANoptosis. Data from the GSE66890 cohort, comprising 29 septic ARDS samples and 28 sepsis control samples, were analyzed to identify 1884 DEGs, with 992 upregulated and 892 downregulated. Volcano plots of DEG expression in the septic ARDS data set are shown in Figure 1A. Due to the stringent nature of adj.P.Val < 0.05, which initially led to no significant DEGs, we adjusted the criterion to P.Value < 0.05 to ensure the identification of relevant genes for further analysis. A relevance score above 3 identified 1355 PRGs in the GeneCards database, including 1313 apoptosis‐related, 11 necroptosis‐related, and 31 pyroptosis‐related genes (see Table S1). Comparison of DEGs from the GSE66890 and GSE32707 cohorts with PRGs revealed 24 overlapping hub genes across the three data sets (Figure 1B). Heatmaps depicting the expression of these 24 DE‐PRGs in the GSE66890 cohort are presented in Figure 1C.

### 3.2 Functional Analysis of Potential Genes

**Figure 2:**
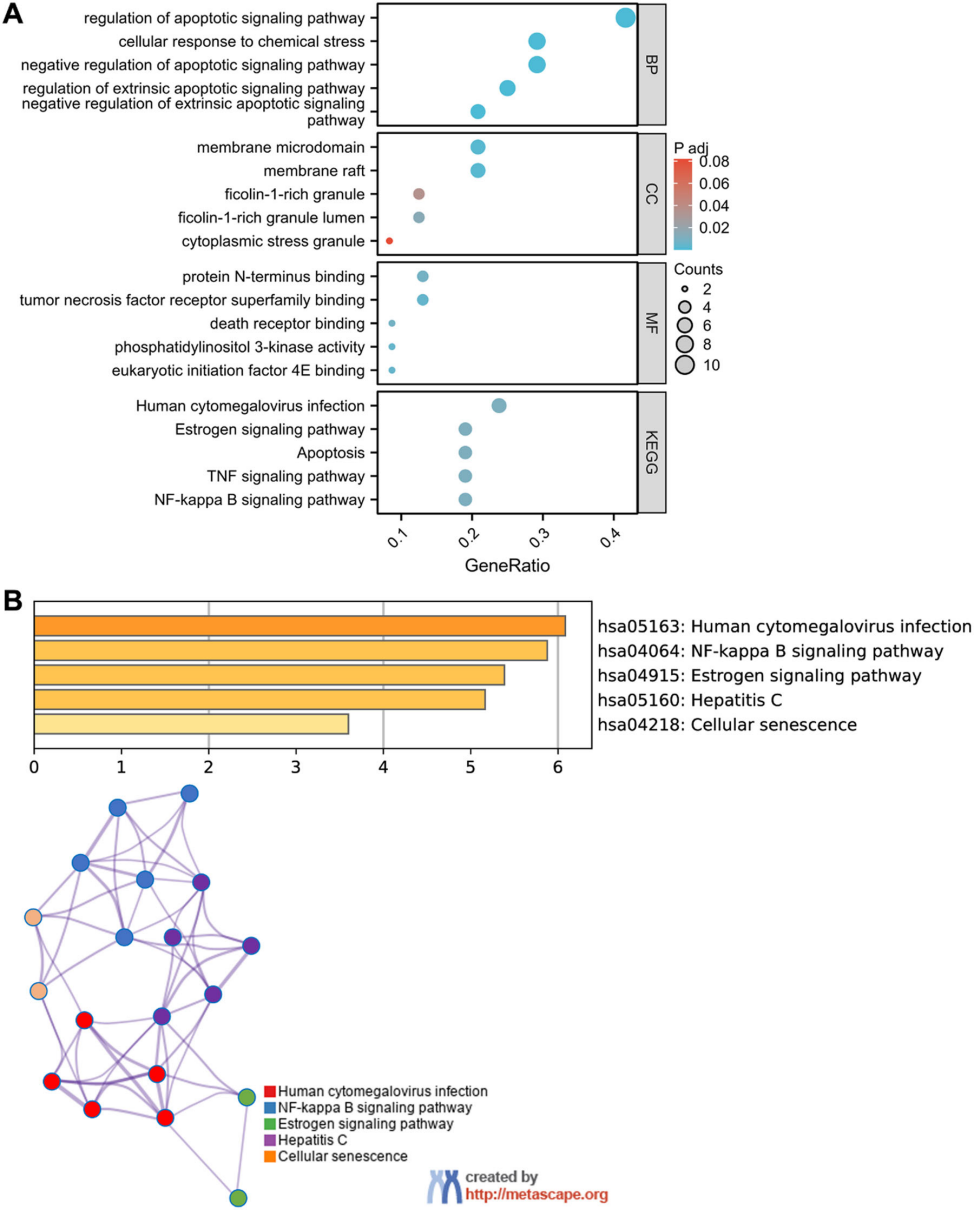
Functional enrichment analysis of differentially expressed genes (DEGs). (A) Gene ontology (GO) analyses highlighting the enriched biological processes (BP), cellular components (CC), and molecular functions (MF), along with pathway enrichment results from the Kyoto Encyclopedia of Genes and Genomes (KEGG). (B) Metascape analysis is presented as a bar chart illustrating non‐redundant enrichment clusters with one representative cluster for each category. Additionally, a network plot shows the relationships among all enriched terms, color‐coded by *p*‐value significance, indicating that terms with a higher gene count tend to exhibit more significant *p*‐values.

To elucidate the biological roles and pathways through which DE‐PRGs may impact the progression of sepsis to ARDS, we conducted GO and KEGG pathway enrichment analyses. These analyses were designed to classify the identified DE‐PRGs into distinct biological processes (BP), cellular components (CC), and molecular functions (MF), thereby offering insights into the underlying mechanisms. The GO analysis indicated that DE‐PRGs were significantly enriched in MF categories such as protein N‐terminus binding and tumor necrosis factor (TNF) receptor superfamily binding, underscoring potential interactions within inflammatory pathways. Figure 2A indicates enrichment of DE‐PRGs in BP terms like “regulation of apoptotic signaling pathways” and “cellular response to chemical stress,” with a strong association with the membrane raft's CC. Additionally, DEGs are enriched in MF categories such as protein N‐terminus binding and tumor necrosis factor receptor superfamily binding. KEGG analysis shows the enrichment of DE‐PRGs in pathways related to NF‐kappa B signaling, apoptosis, infection, and TNF signaling. DEGs are enriched in MF categories like protein N‐terminus binding and TNF receptor superfamily binding. KEGG analysis reveals DE‐PRGs enrichment in pathways related to NF‐kappa B signaling, apoptosis, infection, and TNF signaling. Figure 2A shows Metascape enrichment analysis, but the bar chart lacks clarity on inter‐cluster similarities and intra‐cluster redundancies. In contrast, Figure 2B provides an alternative visualization, showing enrichment in pathways similar to cytomegalovirus infection, cellular response, and estrogen signaling, which may play significant roles in ARDS progression. This suggests that these analogous pathways, including immune responses resembling those of cytomegalovirus infection and estrogen signaling, could be involved in immune dysregulation during ARDS development.

### 3.3 Identification of Key Genes Related to PANoptosis and Development of a Diagnostic Model

**Figure 3:**
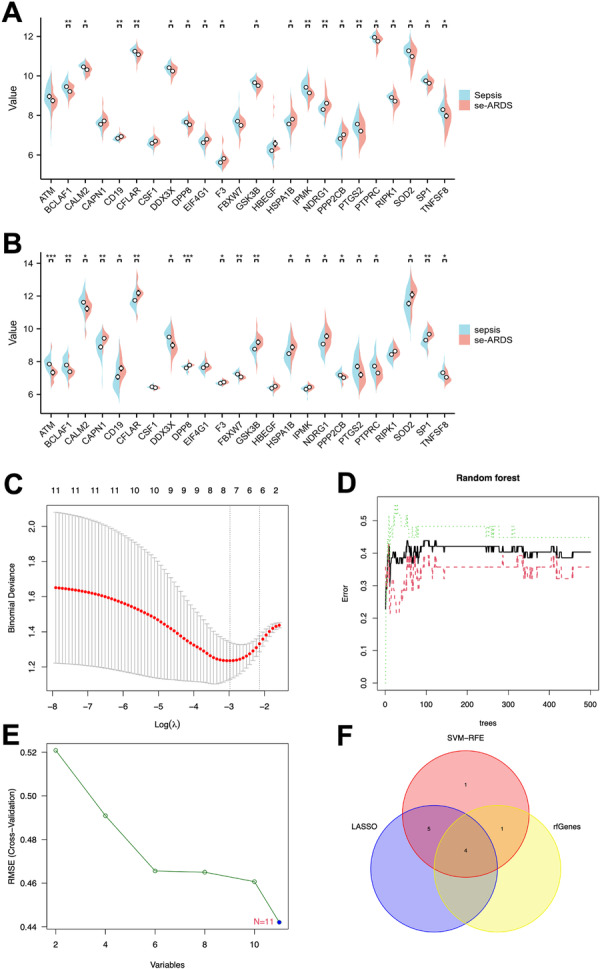
Identification of key genes in septic ARDS. (A) Boxplot illustrating the expression levels of 24 differentially expressed PRGs (DE‐PRGs) in the GSE66890 data set, with blue bars representing sepsis patients and red bars representing septic ARDS patients. (B) Boxplot depicting the expression levels of the same 24 DE‐PRGs in the GSE32707 data set. (C) Identification of diagnostic markers using the least absolute shrinkage and selection operator (LASSO) logistic regression algorithm. (D) Screening of diagnostic markers through the random forest (RF) algorithm. (E) Selection of diagnostic markers via the support vector machine‐recursive feature elimination (SVM‐RFE) algorithm. (F) Venn diagram showing the intersection of four critical variables—NDRG1, DDX3X, PTPRC, and TNFSF8—identified by the LASSO, RF, and SVM‐RFE algorithms. *Significance levels: *p < 0.05; **p < 0.01; ***p < 0.001; ****p < 0.0001.

The goal is to identify key PRGs involved in the progression from sepsis to septic ARDS. By analyzing DE‐PRGs and using machine learning, we aim to identify genes with strong diagnostic potential for septic ARDS, ultimately refining a model for early detection and intervention. We analyzed the GSE66890 and GSE32707 data sets to find intersecting DEGs, identifying 24 that relate to PANoptosis. From these, 11 key genes were consistently differentially expressed and significant across both data sets, including BCLAF1, CALM2, CAPN1, CD19, and DDX3X, F3, HSPA1B, NDRG1, PTGS2, PTPRC, and TNFSF8 (Figure 3A,B). To pinpoint key genes in septic ARDS pathogenesis, we employed multiple machine‐learning algorithms for feature selection. In constructing the ARDS diagnostic model, these 11 PRGs were included. The LASSO regression identified nine significant genes for sepsis‐related ARDS diagnosis (Figure 3C). The RF algorithm highlighted five genes with a mean decrease in Gini > 2 (Figure 3D), while the SVM‐RFE algorithm refined biomarkers to six features, identifying 11 significant genes (Figure 3F). Notably, only four hub genes—DDX3, NDRG1, PTPRC, and TNFSF8—were common across all three methods (Figure 3D).

### 3.4 Construction and Validation of a Diagnostic Model for Septic ARDS

**Figure 4:**
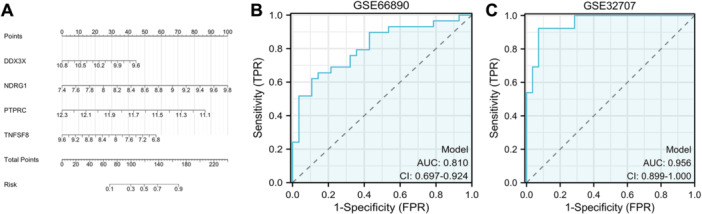
Validation of the model's predictive efficacy. (A) Nomogram representing diagnostic biomarkers for predicting the development of septic ARDS. (B) ROC curve analysis for the GSE66890 training set, demonstrating an AUC of 0.810. (C) ROC curve analysis for the GSE32707 validation set, indicating an AUC of 0.956.

We aim to develop and validate a diagnostic model for septic ARDS by utilizing key genes associated with PANoptosis, thereby providing a dependable tool for early detection and intervention. A nomogram based on logistic regression was constructed using four central hub genes to improve diagnostic and predictive capabilities (refer to Figure 4A). ROC curves evaluated each gene's diagnostic value, with the AUC reflecting their sensitivity and specificity for septic ARDS. The gene prediction model, based on these four genes, exhibited strong performance in the GSE66890 cohort with an AUC of 0.810 (Figure 4B) and was validated in the GSE32707 cohort with an AUC of 0.956 (Figure 4C). This diagnostic model, incorporating five key genes, shows promise for early‐stage septic ARDS prediction.

### 3.5 Pathway Associations of Key PANoptosis Genes in Septic ARDS

**Figure 5:**
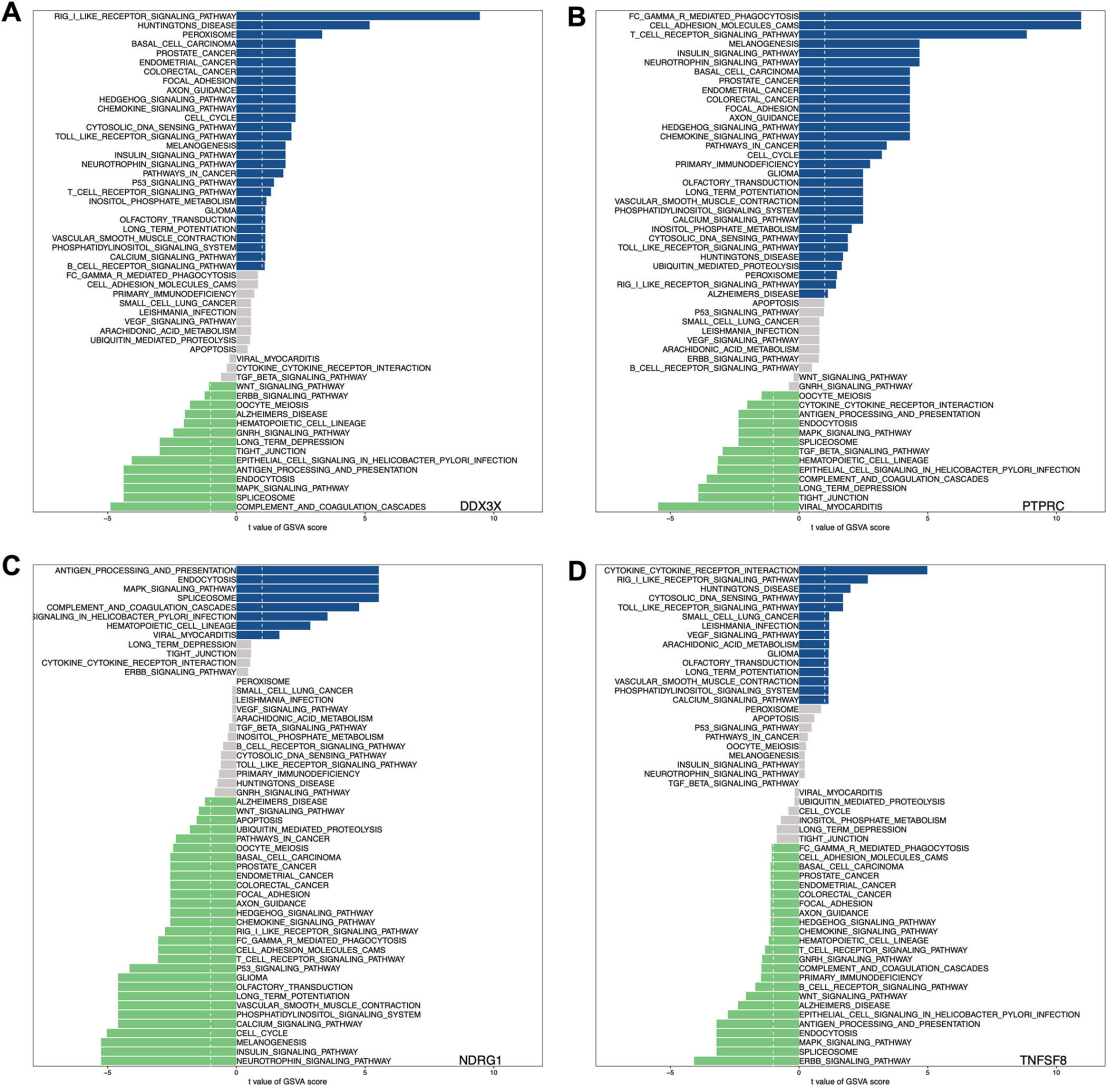
GSVA analysis of critical PRGs. (A−D) Pathway enrichment analysis via GSVA for the key PRGs: DDX3X, PTPRC, NDRG1, and TNFSF8, respectively. Each panel illustrates the specific pathways enriched in association with each gene.

GSVA is a nonparametric, unsupervised approach for assessing signaling pathway activity or gene sets in individual samples. We performed GSVA to investigate the biological importance of critical PRG expression in septic ARDS. Figure 5 highlights the main pathways significantly correlated with PRG expression. NDRG1 expression showed a positive association with the spliceosome, MAPK signaling pathway, endocytosis, antigen processing and presentation, and apoptosis. Conversely, DDX3X, PTPRC, and TNFSF8 expressions were negatively correlated with the spliceosome, MAPK signaling pathway, endocytosis, and antigen processing and presentation.

### 3.6 Landscape of Immune Dysregulation in Septic ARDS Patients

**Figure 6:**
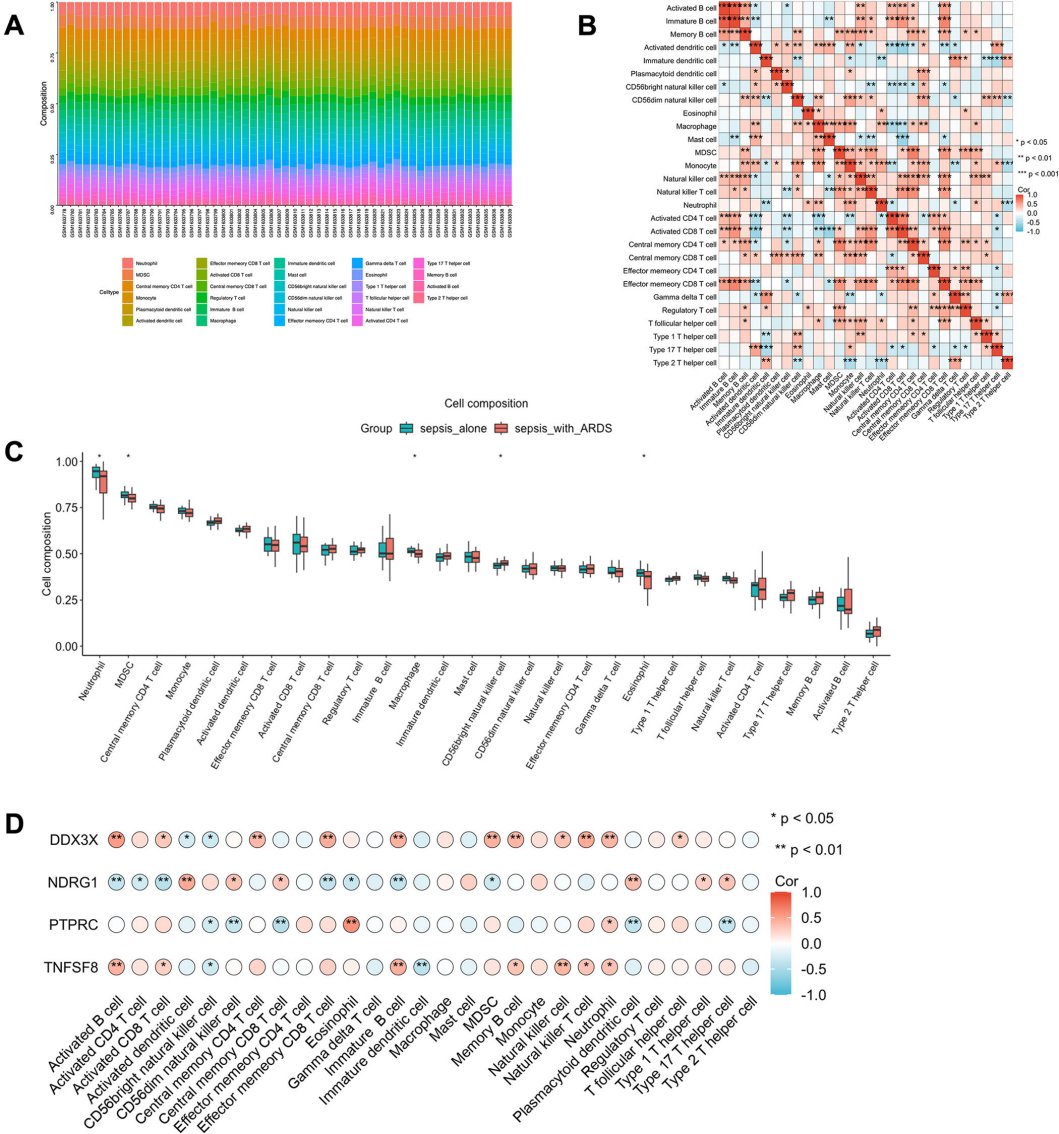
Analysis of immune cell infiltration in septic ARDS. (A) Stacked histogram depicting the proportion of various immune cells within each sample analyzed. (B) Heatmap illustrating the correlations among the proportions of different immune cell types. The intensity of each color indicates the strength of the correlation. (C) Comparative analysis of immune cell type fractions in septic ARDS versus sepsis cohorts. Red indicates the ARDS group, while blue denotes the sepsis group, with statistical *p*‐values displayed above each comparison. (D) Correlation analysis between critical genes and immune cell populations, highlighting potential gene‐immune interactions.

The analysis of sepsis‐related pathogenic ARDS genes indicated a strong link to inflammation and immune processes. We used ssGSEA to analyze immune cell traits and their links to immune regulation, diagnostic biomarkers, and immune cell infiltration in septic ARDS patients. A heatmap was generated to depict immune cell patterns within the sepsis microenvironment. Figure 6A shows the distribution of 28 immune cell types per sample, with notable differences in five subpopulations between septic ARDS and sepsis groups. In sepsis and ARDS, immune cell interactions reveal coordinated responses across activation states and cell types. Activated B cells positively correlate with both immature and memory B cells, suggesting linked activation roles. Activated dendritic cells show strong associations with macrophages, underscoring their role in immune coordination, while immature dendritic cells show fewer connections, pointing to a proinflammatory state. Distinct NK cell subsets, particularly CD56dim cells, correlate with dendritic cells and macrophages, suggesting roles in inflammation regulation. Macrophages, monocytes, and T cells (especially CD4 and CD8 subtypes) demonstrate interconnected responses, emphasizing innate and adaptive immune cell involvement. Regulatory T cells, particularly Type 2 helper T cells, correlate with macrophages and monocytes, highlighting their regulatory function in balancing immune responses in ARDS (Figure 6B). The septic ARDS group had lower proportions of neutrophils, MDSCs, macrophages, CD56‐bright NK cells, and eosinophils compared to the sepsis group (Figure 6C). Further analysis examined the link between four hub gene expressions and immune cell proportions. Figure 6D shows a strong correlation between immune cell accumulation in ARDS and the expression of NDRG1, DDX3X, PTPRC, and TNFSF8.

### 3.7 Regulatory Networks of the Key PRGs in Septic ARDS

**Figure 7:**
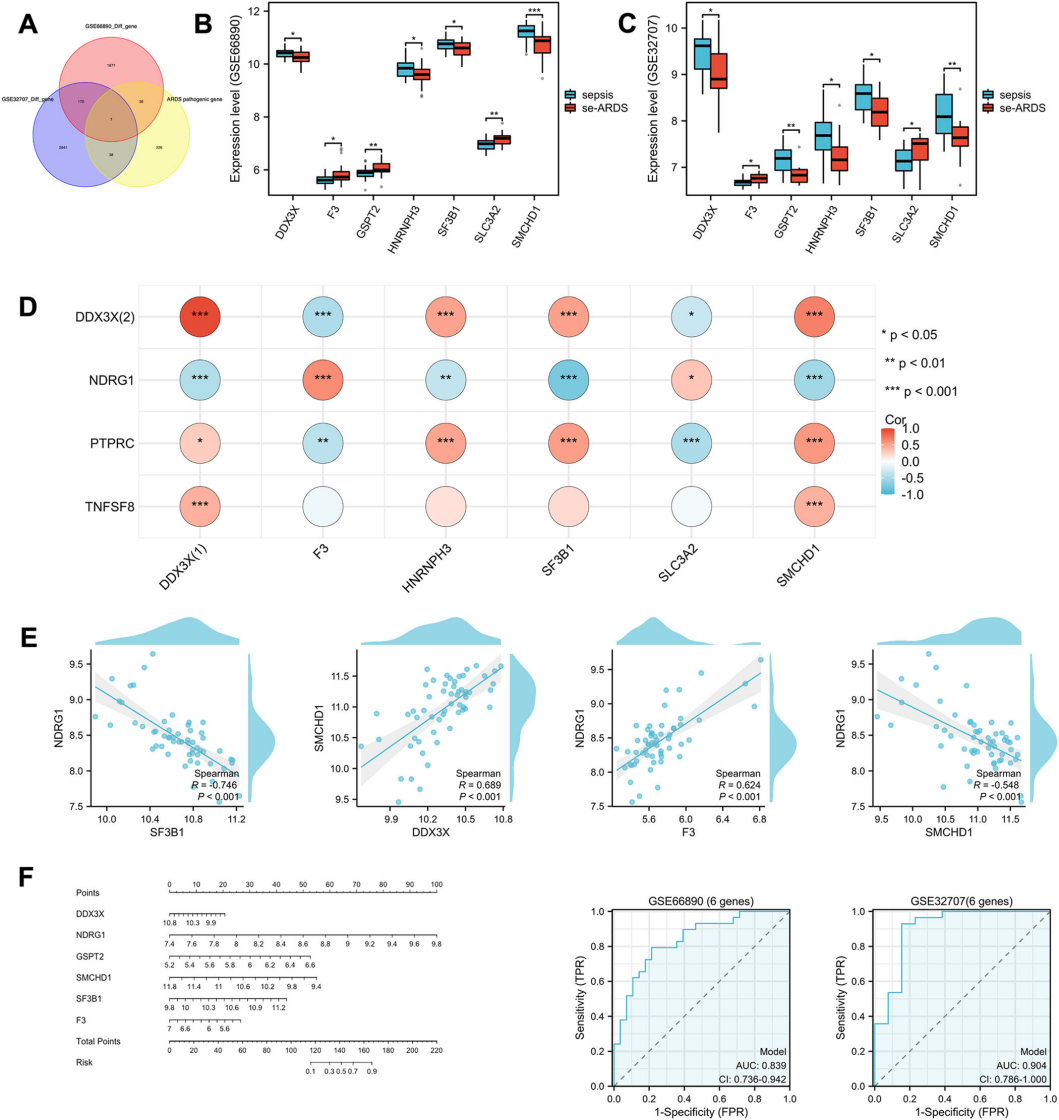
Identification of crucial genes associated with ARDS and their interaction with four pivotal PRGs. (A) Venn diagram illustrating the overlap of differentially expressed genes (DEGs) from studies GSE66890 and GSE32707 with genes known to be related to ARDS. (B) Expression profiles of the intersection genes identified in data set GSE66890. (C) Expression profiles of the intersection genes identified in data set GSE32707. (D) Correlation analyses exploring the relationships between ARDS‐associated genes and four significant PRGs. (E) Detailed correlation analyses among NDRG1, SF3B1, SMCHD1, F3, and DDX3X, emphasizing pairwise interactions. (F) Six‐gene nomogram and ROC analysis for septic ARDS prediction. Significance levels: *p < 0.05; **p < 0.01; ***p < 0.001; ****p < 0.0001.

To investigate the regulatory networks of key PRGs in ARDS progression, we analyzed genes associated with ARDS to find connections with our DE‐PRGs. A total of 307 genes linked to ARDS were identified from the GeneCards database using a relevance score above 1 (see Table S1). Comparison of DEGs from the GSE66890 and GSE32707 cohorts with ARDS‐related genes revealed seven overlapping hub genes (Figure 7A). Notably, six of these genes, including GSPT2, SMCHD1, SF3B1, DDX3X, and F3, showed significant differences and consistent trends between sepsis and septic ARDS samples in the GSE66890 and GSE32707 databases (Figure 7B,C). Pearson's correlation analysis revealed a link between the expression of four key genes and ARDS‐related genes (Figure 7E). NDRG1 showed high differential expression, positively correlating with F3 (*r* = 0.62) and negatively with SF3B1 (*r* = −0.75). The four PRGs also had different correlations with HNRNPH3 and SMCHD1, suggesting they may regulate ARDS by interacting with these pathogenic genes. To assess whether the six genes most strongly linked to septic ARDS can predict ARDS onset, we created an additional nomogram using the six‐gene set (NDRG1, GSPT2, SMCHD1, SF3B1, DDX3X, F3) and compared it to the original four‐gene model. Validation on GSE66890 and GSE32707 data sets showed AUCs of 0.839 and 0.904, indicating comparable diagnostic accuracy (Figure 7F).

### 3.8 Genetic Variants of NDRG1 and Susceptibility to ARDS

**Figure 8:**
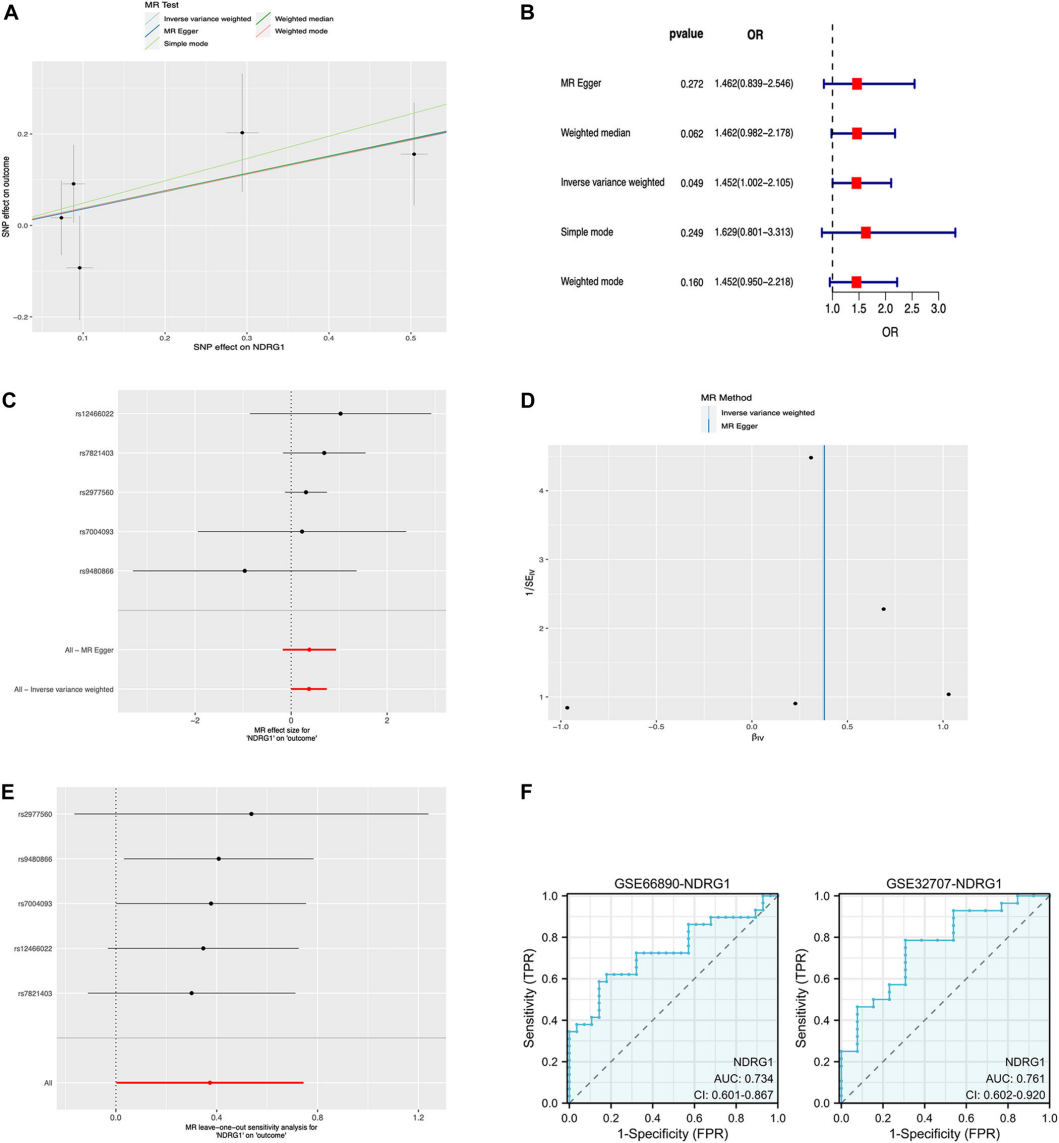
Mendelian randomization analysis of NDRG1 and ARDS. (A) Scatter plots illustrate the causal relationships between NDRG1‐associated SNPs and the risk of ARDS. The slope of each line in the scatter plots represents the causal effect estimated by each method. (B) Summary table of Mendelian randomization (MR) estimates obtained from various methods including inverse‐variance weighted (IVW), weighted median (WM), MR‐Egger, Simple mode, and MR‐PRESSO. (C−E) Visualization of MR analysis results for NDRG1 and ARDS, including forest plots, leave‐one‐out sensitivity analyses, and funnel plots. (F) ROC curve analysis was performed on the GSE66890 training set and the GSE32707 validation set.

Research shows that reducing NDRG1 expression alleviates intestinal damage in septic mice. This study identified NDRG1 as the only gene among four candidates upregulated in septic ARDS. A two‐sample Mendelian randomization analysis examined the causal link between NDRG1 in eQTL and ARDS. After excluding SNPs in linkage disequilibrium and palindromic SNPs, the association between NDRG1 SNPs and ARDS risk was assessed using Mendelian randomization estimates. IVW analysis showed a significant increase in ARDS risk linked to accelerometer‐assessed NDRG1 (OR 1.452, 95% CI 1.002−2.105, *p *= 0.049) (Figure 8A,B). There was no evidence of heterogeneity in both MR Egger and IVW analyses, and the Egger intercept indicated an absence of pleiotropy (Table 1). Furthermore, LOO analysis indicated no single SNPs affected the results, and the symmetric funnel plot supported this conclusion (Figure 8C−E). To evaluate the predictive value of NDRG1 alone for septic ARDS, we performed ROC analysis, which yielded AUCs of 0.734 and 0.761 on the GSE66890 and GSE32707 data sets, respectively. These results further support the strong association between NDRG1 and ARDS onset (Figure 8F).

**Table 1:** Sensitivity and pleiotropy analysis of the causal association between NDRG1 and ARDS.

Exposure	Outcomes	Heterogeneity (MR Egger)		Heterogeneity (IVW)		Pleiotropy (MR‐Egger)	
		*Q* value	*p*	*Q* value	*p*	Intercept	*p*
NDRG1	ARDS	2.35	0.503	2.35	0.671	−0.0021	0.98

Abbreviations: ARDS, acute respiratory distress syndrome; NDRG1, N‐myc downstream regulated 1.

### 3.9 The Validation of the Expression of Hub Genes NDRG1

**Figure 9:**
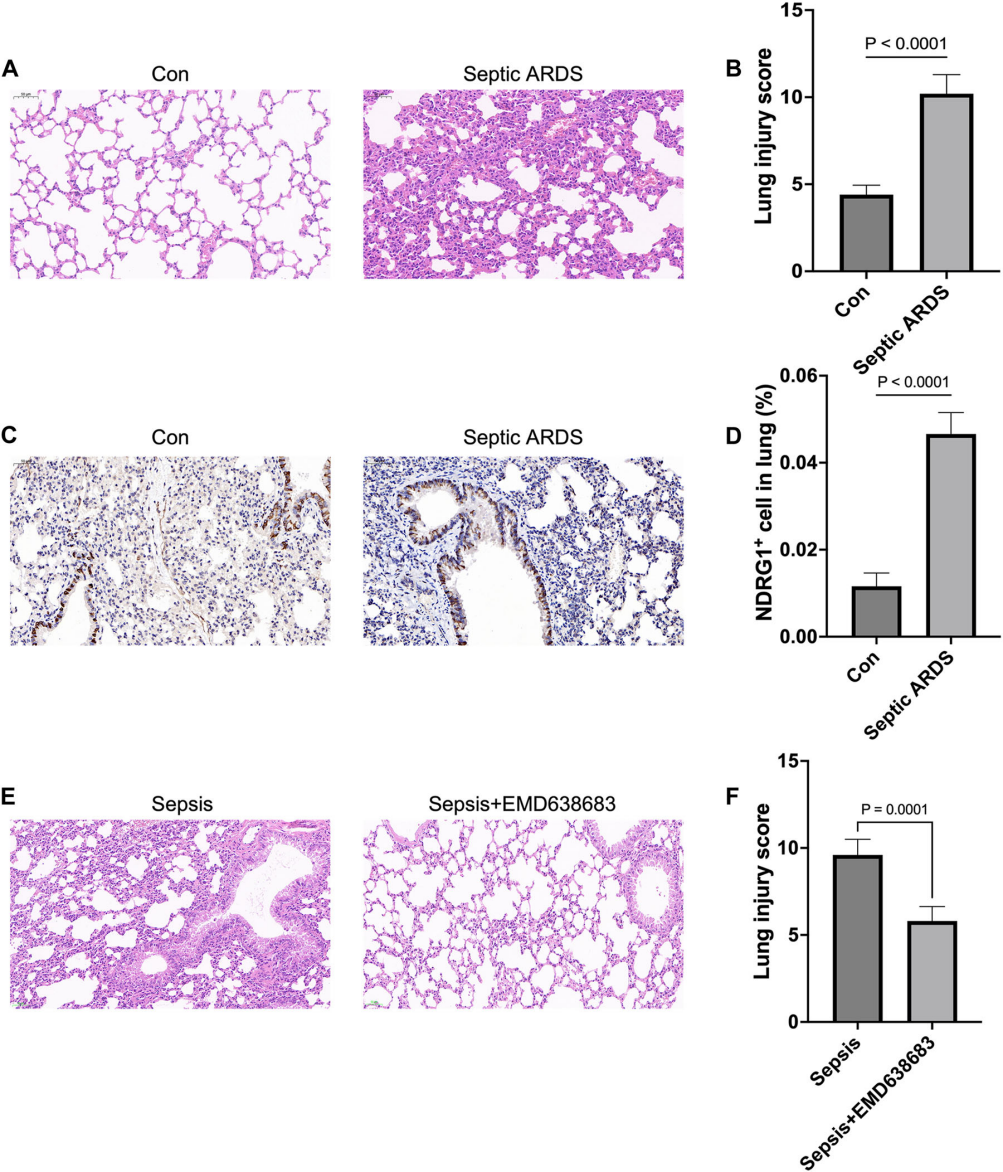
Validation of NDRG1 protein expression. (A) Representative histological sections of lung tissue from ARDS model mice 24 h post‐induction, stained with hematoxylin and eosin (magnification, ×200). (B) Comparative immunohistochemical staining for NDRG1 across different experimental groups. (C, D) Detection and localization of NDRG1 expression in lung tissues of septic ARDS model mice using immunohistochemical staining (magnification, ×200). (E, F) Pathological changes in lung tissue of septic ARDS model mice after NDRG1 inhibition, observed using immunohistochemical staining (magnification, ×200).

In septic ARDS mice, lung injury markers like hemorrhage, edema, inflammatory cell infiltration, and atelectasis were significantly reduced compared to the control group, as shown by lower pulmonary scores (Figure 9A,B). Recent studies underscore the pivotal role of NDRG1 in regulating endothelial inflammation and vascular remodeling, suggesting that its inhibition could enhance therapeutic strategies for inflammatory vascular diseases [24]. Lung endothelial cell injury is a fundamental mechanism in ARDS‐related pulmonary inflammatory edema. Immunohistochemical analysis revealed that NDRG1 is predominantly localized around blood vessels, exhibiting heightened expression in the sepsis ARDS group (Figure 9C,D), accompanied by notable perivascular inflammatory cell infiltration. After NDRG1 inhibitor treatment, septic lung injury was alleviated (Figure 9E,F).

## 4 Discussion

Cell death is essential for physiological homeostasis, and its dysregulation, along with insufficient clearance of dead cells, are significant pathogenic mechanisms in ARDS [25, 26]. Although individual cell death pathways, their mechanisms across cell types, and key molecule expressions in sepsis‐related ARDS have been extensively studied, the complex interactions among these pathways in septic ARDS remain underexplored [27, 28, 29]. However, accumulating evidence indicates that such interactions do occur.

The study found 24 pyroptosis‐related genes with different expressions between septic ARDS and sepsis patients in the GSE66890 data set. Enrichment analyses showed these genes are mainly associated with the MAPK signaling pathway, endocytosis, antigen processing and presentation, and apoptosis. LPS‐induced TLR4/EGFR phosphorylation leads to MAPK14 phosphorylating Rab7a at S72, which disrupts late endocytosis of membrane receptors. This preserves EGFR membrane localization by preventing lysosomal degradation, promotes macrophage M1 polarization, and exacerbates ARDS [30]. The MAPK pathway is crucial in activating PANoptosis, thereby enhancing SAE [31]. The enrichment of pathways related to cytomegalovirus infection, cellular response, and estrogen signaling may play a significant role in the immune dysregulation observed in ARDS. Reactivation of Herpesviridae, particularly in severe cases of ARDS, is frequently associated with prolonged mechanical ventilation and elevated inflammatory biomarkers, which in turn drive cytokine overproduction and contribute to immune imbalance [32]. Thymoquinone, a compound derived from black seed oil, has the potential to mitigate ARDS by modulating inflammation in a manner analogous to its effects in hepatitis C [33]. Additionally, estrogen may attenuate the severity of ARDS in COVID‐19 by reducing ACE2‐dependent NOX2 activation, reactive oxygen species production, and endothelial inflammation [34]. We utilized LASSO, RF, and SVM‐RFE methods to analyze patient clinical characteristics and identified four key differentially expressed potential risk genes associated with septic ARDS: NDRG1, DDX3X, PTPRC, and TNFSF8. The predictive performance of these DE‐PRGs was evaluated with ROC curves, yielding AUCs of 0.810 and 0.956 in two data sets, demonstrating strong and stable predictive value. Their expression profiles were significantly linked to key pathways like MAPK signaling, endocytosis, antigen processing and presentation, and apoptosis, crucial in ARDS development [35, 36, 37].

The NDRG1 gene produces a protein that plays a role in stress and hormonal responses, as well as in cell growth and differentiation. He et al.'s research shows that disrupting NDRG1 hinders DNA damage response and apoptosis in ERCC1‐deficient lung cancer cells when treated with cisplatin/glycopyrrolate sodium [38]. DRG1 plays a crucial role in managing endothelial inflammation, thrombosis, and vascular remodeling [24]. In this study, immunohistochemistry confirmed that NDRG1 is primarily expressed near blood vessels, supporting its involvement in vascular inflammation. Inhibiting NDRG1 expression, as demonstrated with 2% hydrogen (H₂) inhalation, has shown protective effects in septic mice by mitigating intestinal injury [39]. In this study, immunohistochemistry confirmed that NDRG1 is primarily expressed near blood vessels, supporting its involvement in vascular inflammation. Inhibiting NDRG1 expression, as demonstrated with 2% hydrogen (H₂) inhalation, has shown protective effects in septic mice by mitigating intestinal injury [24]. Similarly, after NDRG1 inhibitor treatment, septic lung injury was alleviated, demonstrating its potential to reduce endothelial damage and inflammation. Given the importance of endothelial injury in septic ARDS, NDRG1 may facilitate acute lung injury by promoting vascular endothelial apoptosis and inflammatory damage, positioning it as a potential therapeutic target for inflammatory vascular conditions.

The DDX3X gene encodes a protein with ATP‐dependent RNA deconjugase activity. In sepsis patients, elevated DDX3X mRNA levels in T cells correlate with T‐cell count and APACHE II score [40]. Research by Wang et al. shows that TLR4 triggers microglial pyroptosis after spinal cord injury by activating the DDX3X‐mediated NLRP3 inflammasome via the JAK2/STAT1 pathway [41]. Inhibiting DDX3X can improve inflammation, immune suppression, and catabolism in septic mice [42]. Additionally, decreased DDX3X expression in septic ARDS patients compared to those with sepsis suggests potential immunosuppression. PTPRC [43], also known as CD45, is implicated in apoptosis in ARDS and is a potential therapeutic target for sepsis, with regulation by 4‐octyl itaconate enhancing antimicrobial effects [44, 45, 46]. TNFSF8, crucial for infection and immunity, functions as an apoptosis gene, with its expression in pDMRs predicting anti‐PD‐1 immunotherapy results in small‐cell lung cancer. CD153, encoded by TNFSF8, is essential for regulating CD4 + T cells in tuberculosis and may influence apoptosis control in septic ARDS [47, 48, 49]. The simultaneous interaction and activation of multiple genes involved in PCD support the concept of PANoptosis.

Our analysis revealed that septic ARDS patients had more CD56bright NK cells and fewer neutrophils, macrophages, MDSCs, and eosinophils than sepsis patients. A correlation analysis showed that low expression of DDX3X, PTPRC, and TNFSF8 was significantly positively linked to increased neutrophil infiltration. Neutrophil infiltration in the lungs is a key feature of ARDS following intestinal ischemia/reperfusion, indicating that targeting the NETosis pathway could provide promising therapeutic strategies [50]. Additionally, our findings indicate a negative association between three underexpressed PRGs and CD56bright NK cells. CD56 bright NK cells are capable of producing substantial amounts of interleukin‐10, an immunosuppressive cytokine that has the potential to attenuate autoimmune responses [51]. Eosinopenia is recognized as an accurate early diagnostic indicator for neonatal sepsis [52]. Dexamethasone administration induces immunosuppression in rabbit peripheral blood, evidenced by neutropenia and eosinopenia [53]. The study found eosinophils positively correlated with PRPRC and negatively with NDRG1. Septic ARDS patients exhibited more immunosuppressive tendencies compared to sepsis patients. Investigating the interactions between key PRGs and specific immune cells in sepsis and ARDS is valuable. DDX3X is a pathogenic gene in ARDS and plays a crucial role in PANoptosis. Inhibitors of DDX3X reduce inflammation, maintain body weight and muscle mass, and lower oxidative stress in septic mice, indicating a strong inverse relationship with SMCHD1. Facioscapulohumeral muscular dystrophy 2 (FSHD2) is marked by impaired chromatin compaction due to mutations in SMCHD1 [54]. These findings suggest a link to respiratory muscle injury in ARDS patients.

A limitation of this study is the lack of significant results after multiple testing corrections; to retain a broader set of genes for analysis, we opted not to apply stringent correction, acknowledging the increased risk of false positives. Although the uncorrected results provided insights, they did not maintain statistical significance after correction. To address this, we incorporated additional validation methods, including animal experiments and Mendelian randomization, which supported the involvement of key target genes, particularly NDRG1. However, further clinical validation is necessary to confirm the therapeutic potential of NDRG1 and other identified genes in septic ARDS.

## 5 Conclusion

Our study identified DE‐PRGs—NDRG1, DDX3X, PTPRC, and TNFSF8—in patients with sepsis‐associated ARDS. These genes could be diagnostic markers for predicting ARDS in sepsis patients, with NDRG1 showing a causal link to ARDS. Our findings suggest that PANoptosis may contribute to immune dysregulation in sepsis‐related ARDS, positioning NDRG1 as a potential target for developing ARDS treatments.

## Author Contributions

Zhong‐Hua Lu and Yun Sun participated in the screening and assembly of the data set and the analysis and interpretation of the data. Yan Tang, Mei Liu, Lu Fu, and Hu Chen participated in the topic discussion and data statistical analysis. Hu Chen, Xian‐Kai Wang, and Ming‐Juan Li participated in immune infiltration analysis and Mendelian randomization analysis. Feng Liu, Wei‐Li Yu, and Yan Tang contributed to the R statistical analysis of the study. Xian‐Kai Wang and Zhong‐Hua Lu took part in the animal experiments. All authors participated in the experiment and drafted the manuscript together.

## Ethics Statement

Ethical approval was granted by the Ethics Committee of Anhui Medical University.

## Conflicts of Interest

The authors declare no conflicts of interest.

## Supporting information

Supporting information.

Supporting information.

Supporting information.

## Data Availability

All raw data and code are available upon request.
