# Supplementary figures and images for: Identification and Functional Analysis of PANoptosis‐Associated Genes in the Progression From Sepsis to ARDS
Source: Immun Inflamm Dis. 2025 Jan 24;13(1):e70136. doi: 10.1002/iid3.70136 (PMC11760491; doi:10.1002/iid3.70136)

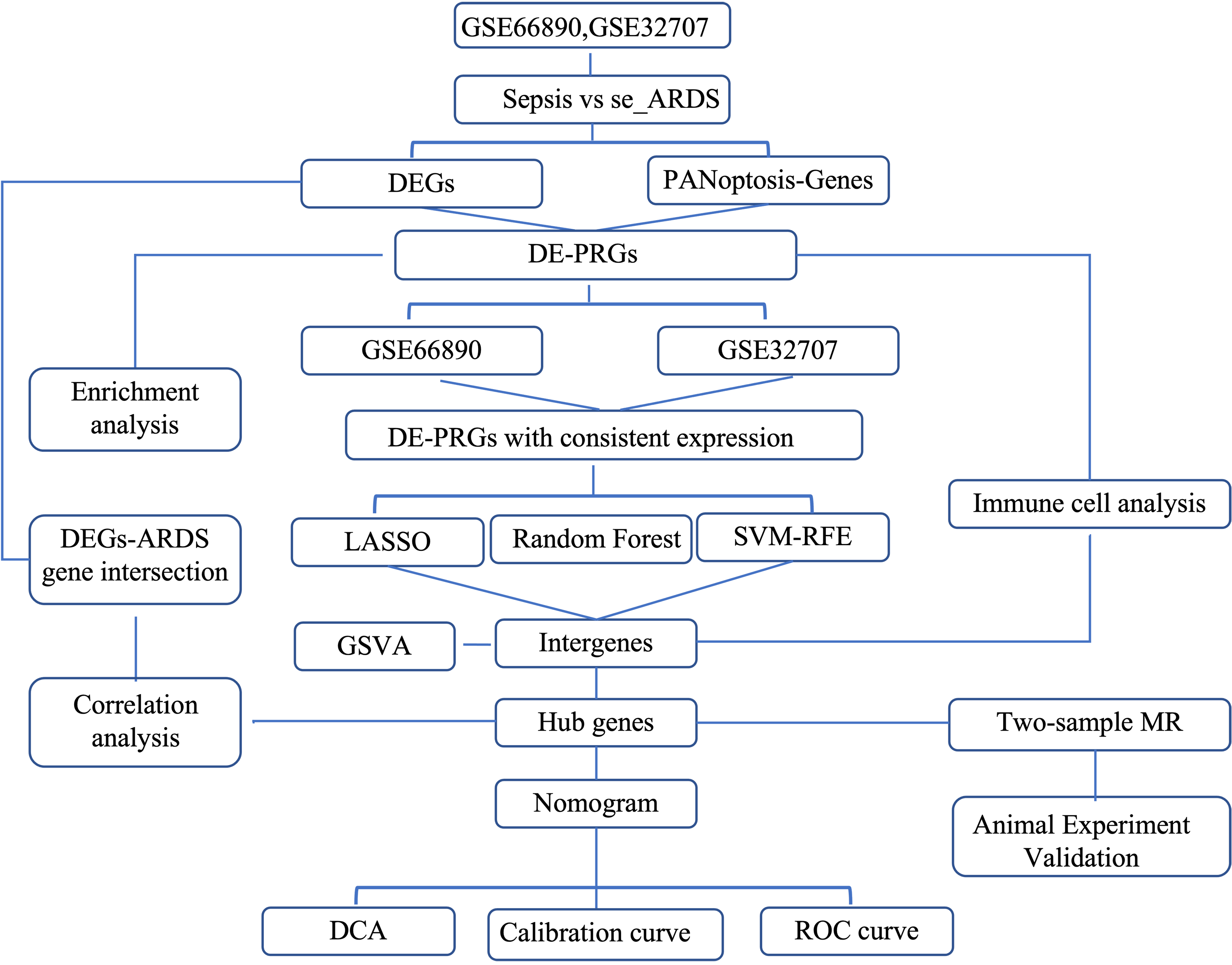

Supplement: Supplementary file 2 — Supporting information. [file IID3-13-e70136-s003.tif]
